# Primary thyroid lymphoma: a case of postoperative diagnosis in a patient with toxic multinodular goiter

**DOI:** 10.1097/j.pbj.0000000000000246

**Published:** 2024-03-08

**Authors:** Margarida Nunes Coelho, Filipe Cunha, Joana Isabel Almeida, Tatiana Santos, Isabel Marques

**Affiliations:** a General Surgery Department of Centro Hospitalar do Tâmega e Sousa, Penafiel, Portugal; b Endocrinology Department of Centro Hospitalar do Tâmega e Sousa, Penafiel, Portugal

## Introduction

Thyroid cancer is one of the most frequent malignancies worldwide, corresponding to 1% of all malignant neoplasms.^1^ Contrarily, primary thyroid lymphoma (PTL) is extremely rare, representing only 2–5% of all thyroid cancers.^1,2^ PTL is more prevalent in women, with a female-to-male ratio of 3:1, and it presents typically in the 6th and 7th decades of life.^1^

Practically, all PTLs are non-Hodgkin lymphomas (NHLs), and the most common NHL subtype is the diffuse large-cell lymphoma (DLBCL).^3,4^ The pathogenesis of PTL remains unclear. Because there is no native lymphoid tissue in the thyroid gland and Hashimoto thyroiditis (HT) is a known risk factor of PTL, this led to the hypothesis that persistent antigenic stimulation in HT and consequent chronic proliferation of the lymphoid tissue increases the risk of cell mutations and lymphoma development.^1,2,4,5^

The most common form of presentation is a painless, rapidly growing goiter, with or without cervical compression or B symptoms. Most patients are euthyroid at presentation, but hypothyroidism is frequent given its association with Hashimoto thyroiditis. Hyperthyroidism is rarer, but there have been some reported cases of PTL in patients with Graves disease. As far as our literature review could find, there was only a few cases of reported association between PTL and hyperthyroidism and none associated with toxic nodular goiter.^1,6^

We present a case of a DLBCL discovered incidentally after total thyroidectomy in a woman with hyperthyroidism due to toxic multinodular goiter.

## Case Report

We present a case of a 64-year-old woman who presented with weight loss (12 kg in 8 years) and generalized hyperhidrosis without night sweats. She had no other symptoms suggestive of thyrotoxicosis, no compressive symptoms, and no other B symptoms. She had a history of type 2 diabetes mellitus with good glycemic control and no chronic complications, dyslipidemia, and bilateral carpal tunnel release surgery. She was medicated with metformin 1000 mg twice daily, sitagliptin 100 mg daily, and simvastatin 20 mg daily. She was a non-smoker and had no history of cervical irradiation. Her sister had a history of unspecified thyroid disease.

Her thyroid stimulating hormone (TSH) was <0.005 µIU/mL (normal range: 0.38–5.33 µIU/mL) and a previous TSH <0.005 µIU/mL five years ago. Free T4 was 18.3 pmol/L (12–30 pmol/L), and free T3 was 2.78 (2.5–4.4 pg/mL). Her anti-thyroperoxidase (TPOAb) and anti-thyroglobulin (TgAb) antibodies were negative. Thyroid ultrasound revealed an enlarged gland with multiple bilateral thyroid nodules: two dominant nodules in the left lobe measuring 37 × 36 mm and 41 × 26 mm and one 18 × 10 mm nodule in the right lobe, all with microcalcifications classified as EUTIRADS 5. No suspicious lymph nodules were detected. Her previous fine-needle aspiration cytology of a 45-mm nodule in the left thyroid lobe was benign (Bethesda II). The thyroid Tc-99m scintigraphy showed increased thyroid uptake [9.8% (normal range 0.4–4.0%)] with heterogeneous uptake in the left thyroid lobe and diminished uptake in the right lobe.

The diagnosis of toxic multinodular goiter was made, and methimazole was started. She was proposed to undergo total thyroidectomy that was uneventful.

The histological analysis of the surgical specimen showed multiple thyroid nodules in both lobes with benign thyroid hyperplasia. In the right lobe, a nodule measuring 1.7 × 1.1 × 2.0 cm was seen, well-defined, brownish, and partially encapsulated, microscopically with a large cell lymphoid proliferation that infiltrates part of the hyperplasic nodule. The immunohistochemistry profile displayed positivity for CD20, CD45, Bcl2, Bcl6, and MUM 1 and CD10, CD5, cyclin D1, thyroglobulin, and CAM5.2 negativity. These findings were compatible with the diagnosis of DLBCL with activated B-cell like.

Staging fluorodeoxyglucose (FDG)-positron emission tomography (PET) and bone marrow biopsy revealed no signs of lymphoma in activity.

Our patient was diagnosed with DLBCL localized to the thyroid gland, stage IE disease, and underwent three cycles of chemotherapy with rituximab-cyclophosphamide-doxorubicin-vincristine-prednisone (RCHOP) and currently has no evidence of disease.

## Discussion

PTL is a rare malignancy and typically occurs in middle-aged individuals, predominantly women.^4^ The most common type of PTL is a DLBC lymphoma, accounting for up to 70% of cases. DLBC has an aggressive subtype, frequently with metastases at presentation, and is classically associated with poor prognosis. The overall prognosis of PTL has been described as generally excellent, but is subtype-dependent.^1,7^

The main symptoms at presentation are rapid painless thyroid enlargement and cervical compression symptoms such as hoarseness, dyspnea, and dysphagia. Some patients also present with night sweats, fever, and weight loss.^4,7^ Our patient, on the contrary, showed no symptoms of cervical compression, and the gradual weight loss (12 kg in 8 years) and sweating at might were more easily explained by the hyperthyroidism.

The main risk factor of PTL is the presence of Hashimoto thyroiditis, and that explains the frequent association with hypothyroidism.^8^ Some cases of hyperthyroidism were also reported, mainly in patients with Graves disease or in anti-TSH receptor antibody (TSHrAb)-negative patients with no thyroid scintigraphy, making a final etiological diagnosis difficult.^1,4^

The association between Graves disease and PTL is plausible and follows the same hypothesis that chronic antigenic stimulation of the thyroid gland results in the migration of lymphoid tissue to the gland and consequent risk of cell mutation and neoplasia.^1,4^

In our patient, hyperthyroidism was not caused by Graves disease as proven by the thyroid scintigraphy. The TPOAb and TgAb were negative, and we did not measure the TSHrAb.^9^ As far as our literature review could find, there was no previous report of PTL found incidentally in a patient with toxic multinodular goiter and negative autoimmunity.

As in our patient, most cases of PTL are diagnosed retrospectively after surgical excision of the thyroid gland for other causes.^10^

PTL treatment depends on the histological subtype and stage of disease.^4^ The role of surgery has changed. Primary surgery does not seem to improve the outcome, and patients who underwent medical treatment had better prognosis than those with surgical excision and adjuvant chemotherapy.^1,4,7^ Nowadays, the role of surgery is diagnostic or for symptomatic relief. The gold standard of treatment is chemotherapy using a combination of RCHOP with or without radiotherapy. The prognosis of PTL is subtype-dependent. In general, it is described as excellent in many series of cases.^1,2^

The largest series of cases of thyroid lymphoma in the literature are summarized in Table 1.

**Table 1:** Summary of selected original publications, addressing thyroid lymphoma

Study	Type of study	Countries	Number of patients included	Type of PTL	Relevant conclusions
Hirokawa et al^6^ (2020)	Retrospective	Japan, Turkey, Portugal, Taiwan, Thailand, Serbia, China, India	153	MALT 54.9% DLBC 38.6% Other 6.5%	PTL incidence is higher in Western countries Hashimoto thyroiditis is a risk factor of PTL US, biopsy and immunohistochemistry are essential for diagnosis
Onal et al^8^ (2011)	Retrospective	Turkey, China, Netherlands, United States, Greece, Switzerland	87	DLBC 64% MALT 21% Follicular 10% Other 5%	The majority had thyroid enlargement and presented in stage I or II at diagnosis DFS, OS, and LC rates were better in patients with thyroiditis with MALT Age, B-symptoms, lymph node involvement and treatment modality are independent prognostic factors
Noble et al^10^ (2019)	Retrospective	United States	3466	DBCL 59.8% MALT 18.3% Follicular 8% Burkitt 1.9%	PTL are rare and heterogeneous in presentation and histology Most common treatment is multiagent chemotherapy Overall prognosis of PTL is good
Yi et al^7^ (2020)	Retrospective	China	58	DBCL	Optimal treatment methods for DBCL are not established Combination of chemotherapy and radiotherapy had better prognosis Surgical treatment alone is only used for diagnosis
Doria et al^5^ (1994)	Retrospective	United States	11	DBCL (8/11)	Combined modality treatment with surgery, CT, and RT significantly reduces distant and overall recurrence in PTL
Thieblemont et al^4^ (2002)	Retrospective	France	26	DBCL 50% MALT 23% Follicular 12% Burkitt 4% SLL 4%	Most patients have a history of autoimmune thyroiditis With total thyroidectomy, prognosis was good No clinical or biological parameters that could predict evolution of Hashimoto thyroiditis to MALT were found
Yokozawa^2^ (1998)	Retrospective	Japan	155	-	Association of PTL with Hashimoto thyroiditis in 68% PTL occurs in older age compared with differentiated thyroid carcinomas FNAC was diagnostic in 90% of cases PTL treatment is not well established; a combination of surgery, RT, and CT is possible

CT, chemotherapy; DLBC, diffuse large B-cell lymphoma; DFS, disease-free survival; FNAC, fine-needle aspiration cytology; LC, local control; MALT, mucosa-associated lymphoid tissue lymphoma; OS, overall survival; PTL, primary thyroid lymphoma; RT, radiotherapy; SLL, small lymphocytic lymphoma; US, ultrasound.

This case report aims to raise awareness about this rare diagnosis and points out that a patient who presents with hyperthyroidism regardless of the etiology may harbor a PTL, especially in the context of rapidly growing thyroid mass and in the presence of B symptoms. A high level of suspicion is necessary to establish this diagnosis to avoid unnecessary surgery and to improve the overall survival.
